# Transmission of sheep-bovine spongiform encephalopathy to pigs

**DOI:** 10.1186/s13567-015-0295-8

**Published:** 2016-01-07

**Authors:** Carlos Hedman, Rosa Bolea, Belén Marín, Fabien Cobrière, Hicham Filali, Francisco Vazquez, José Luis Pitarch, Antonia Vargas, Cristina Acín, Bernardino Moreno, Martí Pumarola, Olivier Andreoletti, Juan José Badiola

**Affiliations:** Veterinary Faculty, Centro de Investigación en Encefalopatías y Enfermedades Transmisibles Emergentes (CIEETE), Universidad de Zaragoza, 50013 Zaragoza, Spain; Veterinary Hospital, Universidad de Zaragoza, 50013 Zaragoza, Spain; Veterinary Faculty, Department of Animal Medicine and Surgery, Universitat Autònoma de Barcelona, 08193 Barcelona, Spain; UMR INRA ENVT 1225, Interactions Hôtes Agents Pathogènes, Ecole Nationale Vétérinaire de Toulouse, 31076 Toulouse, France

## Abstract

Experimental transmission of the bovine spongiform encephalopathy (BSE) agent has been successfully reported in pigs inoculated via three simultaneous distinct routes (intracerebral, intraperitoneal and intravenous). Sheep derived BSE (Sh-BSE) is transmitted more efficiently than the original cattle-BSE isolate in a transgenic mouse model expressing porcine prion protein. However, the neuropathology and distribution of Sh-BSE in pigs as natural hosts, and susceptibility to this agent, is unknown. In the present study, seven pigs were intracerebrally inoculated with Sh-BSE prions. One pig was euthanized for analysis in the preclinical disease stage. The remaining six pigs developed neurological signs and histopathology revealed severe spongiform changes accompanied by astrogliosis and microgliosis throughout the central nervous system. Intracellular and neuropil-associated pathological prion protein (PrP^Sc^) deposition was consistently observed in different brain sections and corroborated by Western blot. PrP^Sc^ was detected by immunohistochemistry and enzyme immunoassay in the following tissues in at least one animal: lymphoid tissues, peripheral nerves, gastrointestinal tract, skeletal muscle, adrenal gland and pancreas. PrP^Sc^ deposition was revealed by immunohistochemistry alone in the retina, optic nerve and kidney. These results demonstrate the efficient transmission of Sh-BSE in pigs and show for the first time that in this species propagation of bovine PrP^Sc^ in a wide range of peripheral tissues is possible. These results provide important insight into the distribution and detection of prions in non-ruminant animals.

## Introduction

Transmissible spongiform encephalopathies (TSE) are chronic neurodegenerative disorders that affect humans and animals and are associated with the accumulation of an abnormal isoform (PrP^Sc^) of the cellular prion protein (PrP^C^) in the central nervous system (CNS) [1]. TSE are characterized by spongiform changes in the grey matter accompanied by astrocytosis and microgliosis [2–4]. The new variant of Creutzfeldt-Jakob disease (nvCJD) in humans [5] has been linked with the consumption of bovine spongiform encephalopathy (BSE) contaminated meat or meat products during the BSE epidemic in the UK and elsewhere. Moreover, one BSE natural case in a goat in France [6] and another one in the UK [7, 8] have been reported. Sheep and goats can also be experimentally infected using homogenized brain from affected animals as inocula [9–11]. While BSE infection is largely restricted to the nervous system in cattle [12, 13], PrP^Sc^ is widely distributed in the lymphoid tissues of sheep experimentally infected with BSE [10, 14], suggesting that infected sheep could constitute a secondary and more dangerous source of BSE infection for other species, including humans [15–17].

TSE has not been reported in natural conditions in pigs [18], and there is no evidence of BSE transmission between pigs fed with brain material from cattle [19]. However, despite the existence of a strong transmission barrier, signs of TSE have been reported in pigs challenged simultaneously with BSE-derived material via intraperitoneal, intravenous and intracerebral administration [20–22]. Those studies demonstrated pathological changes and PrP^Sc^ deposition in the CNS, but reported no evidence of PrP^Sc^ distribution in other organs. Given the possible lifting of the European Union’s ban on feeding pigs and poultry with animal meal, it is vital that TSE transmission be studied in supposedly resistant species, such as swine, that form part of the human food chain. Pigs are the source of a wide range of food products, and pork is one of the most widely eaten meats in the world. Blood is frequently collected during slaughter for blood sausage production and natural sausage casings are almost exclusively prepared from different parts of the alimentary tract of pigs. The use of pigs as graft donors is also a cause for concern, given a reported case of CJD type 1 in a recipient of a porcine dura-mater graft [23]. It has also been demonstrated that BSE experimentally passaged in sheep (Sh-BSE) homozygous for the A_136_R_154_Q_171_ allele of ovine prion protein (PrP) exhibits altered pathobiological properties due to a decreased polymorphism barrier [24]. The virulence of Sh-BSE in transgenic mice expressing porcine [15] and human PrP [16, 25] is enhanced with respect to the original cattle BSE prion isolate.

This study is the first to describe the tissue distribution of PrP^Sc^ in pigs experimentally infected with BSE previously passaged in sheep, as well as the clinical and neuropathological consequences.

## Materials and methods

### Ethics statement

All procedures were carried out under Project License COTSA EFA 85/08 and CONCOTSA EFA 205/11 and were approved by the in-house Ethic Committee for Animal Experiments under license PI 13/10 from the University of Zaragoza. All animal experiments were performed in accordance with the Spanish Policy for Animal Protection RD1201/05 and European Union Directive 86/609 for the protection of animals used for experimental and other scientific purposes.

### Sheep BSE inoculum

Sheep BSE isolate (Sh-BSE) was originally derived from a pool of ARQ/ARQ sheep that were experimentally infected by intracerebral inoculation with the BSE agent [26]. This isolate was supplied by the Institut National de la Recherche Agronomique (INRA-Toulouse, France).

### Experimental challenge of pigs with sheep BSE

Eight 8-month old minipigs (1 castrated male and 7 females) from the Instituto Madrileño de Investigación y Desarrollo Rural, Agrario y Alimentario (IMIDRA), were intracerebrally inoculated under general anesthesia. Seven animals were challenged with 0.5 mL of inoculum consisting of a 10% homogenate of the Sh-BSE agent in sterile saline solution, administered in a single injection. One animal was challenged with 0.5 mL of sterile saline solution. The injection site was located 1 cm lateral to the midline in the frontal region. The trephine was performed with a dental drill and the inoculate administered via a 20G × 2 ¾” needle.

### Clinical monitoring

Pigs were monitored daily by animal husbandry staff, and 1–7 veterinary clinical assessments were carried out per week, depending on the stage of the animals.

### Tissue sample collection

Animals were euthanized by exsanguination after intravenous pentobarbital injection (DOLETHALND^®^; 10 mg/kg), which was administered on observation of clinical signs suggesting an encephalopathy or the presence of a life-threatening or welfare-compromising disease. Necropsies were conducted systematically and samples collected from the central nervous system (CNS), peripheral nervous system (PNS), lymphoreticular system (LRS), gastrointestinal tract (GIT), skeletal muscles and other tissues. In all cases, tissues samples were collected in duplicate; one sample was stored at −80 °C and the other in 10% formal saline solution.

### Histopathology and immunohistochemistry

For detailed CNS studies, transverse sections of the following areas were selected and stained with hematoxylin and eosin (H&E): frontal cortex (FC), basal ganglia (BG), lateral frontal cortex (LFC), thalamus (T), hypothalamus (Ht), temporal/parietal cortex (TPC), hippocampus (HC), occipital cortex (OC), mesencephalon (Ms), cerebellum (Cbl), 4 neuronal nuclei [the hypoglossal motor nucleus (HMN); the nucleus of the trigeminal nerve spinal tract (NTN); the olivary nucleus (ON); and the dorsal motor nucleus of the vagus nerve (DMNV)], the reticular formation (RF) of the medulla oblongata (MO), and the cervical (CSC), thoracic (TSC), and lumbar spinal cord (LSC). PrP^Sc^ detection was performed in adjacent sections following pretreatment with 98% formic acid, hydrated and autoclaved to enhance antigen retrieval. After proteinase K digestion (4 g/mL), the sections were incubated with blocking reagent (DAKO) for 10 min to block endogenous peroxidase activity, as previously described [27]. Next, sections were incubated with the monoclonal primary antibody 2G11 (1:400) [28] at room temperature (RT) for 1 h. The study of the PrP^Sc^ deposition types and distribution pattern were based on the descriptions reported in natural and experimental scrapie, and experimental BSE in sheep [29–31].

Astrocytosis was evaluated using glial fibrillary acidic protein (GFAP) immunostaining, as previously described [32], and microglia identified in FC, BG, T, Hc, Cbl, MO and CSC sections by immunohistochemical detection of the active form of a calcium-binding protein, specially expressed in microglia cells (Iba-1). After heat-induced epitope retrieval by pretreatment with citrate buffer (pH 6.0), sections were incubated for 1 h at RT with the primary anti-GFAP antibody (rabbit polyclonal, 1:500; DAKO) and overnight at 4 °C with Iba-1 (goat polyclonal 1:600; Abcam), respectively. Sections were subsequently counterstained with hematoxylin.

### Western blotting

Samples were treated with proteinase K, subjected to sodium dodecyl sulfate polyacrylamide gel electrophoresis (SDS-PAGE), and blotted onto membranes according to the Prionics AG (Schlieren, Switzerland) Check^®^ Western BSE test protocol [33], using Sha31 monoclonal antibody (1:8000; Spibio A03212), the Bio-Rad VersaDoc imaging system and Quantity One 1-D Analysis Software (Bio-Rad) for visualization.

### IDEXX HerdChek^®^ BSE-Scrapie Antigen Test

A ligand-based enzyme immunoassay (IDEXX HerdChek^®^ BSE-Scrapie Antigen Test, hereafter referred to as IDEXX), which does not use a PK digestion step, was used to analyze a wide number of samples according to the manufacturer’s instructions, using the conjugate for cattle in each sample. Samples (300 mg) were processed as previously described for sheep tissues [34]. The negative cut-off value applied for the bovine conjugate was 0.14 absorbance units.

### Data analysis

Vacuolation, PrP^Sc^ deposition, astrocytosis and microgliosis in the CNS were evaluated in the stained sections and scored on a scale ranging from 0 to 5 (0 = not detectable; 1 = occasional; 2 = mild; 3 = moderate; 4 = severe; 5 = extreme). A possible correlation between lesions and immunolabeling in the CNS was evaluated using the non-parametric Spearman’s rho measure. *P* < 0.05 was considered significant, *P* < 0.01 very significant and *P* < 0.001 highly significant. Lesion and immunolabeling scores were plotted as a function of the anatomical area, and data expressed as the mean ± standard deviation.

In peripheral tissues, positive results were scored according to the intensity of PrP^Sc^ labeling, and were classified as negative (−), minimal-to-mild (+), moderate (++) or strong (+++).

## Results

Details of incubation period, date of euthanasia, clinical signs and PrP^Sc^ detection by IHC and WB in the brains of all animals are provided in Table 1.

**Table 1:** **Clinical and pathological features.**

Animal id.	Incubation period (weeks)	Euthanasia date (weeks)	Clinical Signs	NPC	PrP^Sc^	PrP^res^
P-1	96	116	Present-Dull form	Present	Present	Present
P-2	109	132	Present-Dull form	Present	Present	Present
P-3	96	115	Present-Dull form	Present	Present	Present
P-4	109	131	Present-Dull form	Present	Present	Present
P-5	77	90	Present-Dull form	Present	Present	Present
P-6	109	131	Present-Aggressiveness	Present	Present	Present
P-7		42	Absent	Absent	Absent	Absent
P-8				Absent	Absent	Absent

Animal identification number, incubation period, euthanasia date, clinical signs, neuropathological changes (NPC) as determined by hematoxylin-eosin (HE) staining, presence of PrP^Sc^ (immunohistochemistry) and presence of PrP^res^ in CNS samples (Western blot).

### Clinical signs and disease course

One pig (P-7) was euthanized for preclinical analysis at 42 weeks post-injection (wpi). The remaining inoculated animals developed clinical signs between 77 and 109 wpi [100 ± 12.9 wpi, mean and standard error of the mean (SEM)]. Behavioral changes were the first clinical signs observed. Animals became highly apprehensive and over-reactive, squealing loudly and fleeing in apparent panic whenever approached or touched. Subsequent behavioral changes suggested confusion in all pigs and depression in 5, which showed listlessness. P-6 showed apparent confusion and anxiety that increased with the progression of the disease. Behavioral changes were accompanied by initially mild hind-limb ataxia followed by progressive locomotor disability with generalized ataxia of gait, weakness and other movement disorders in all pigs. Pigs showed low carriage of the head and ears and signs of tremor in the shoulder regions, flanks and the ears. Persistent recumbency with difficulty rising was also observed.

### Histopathology and immunohistochemistry of the CNS

**Figure 1:**
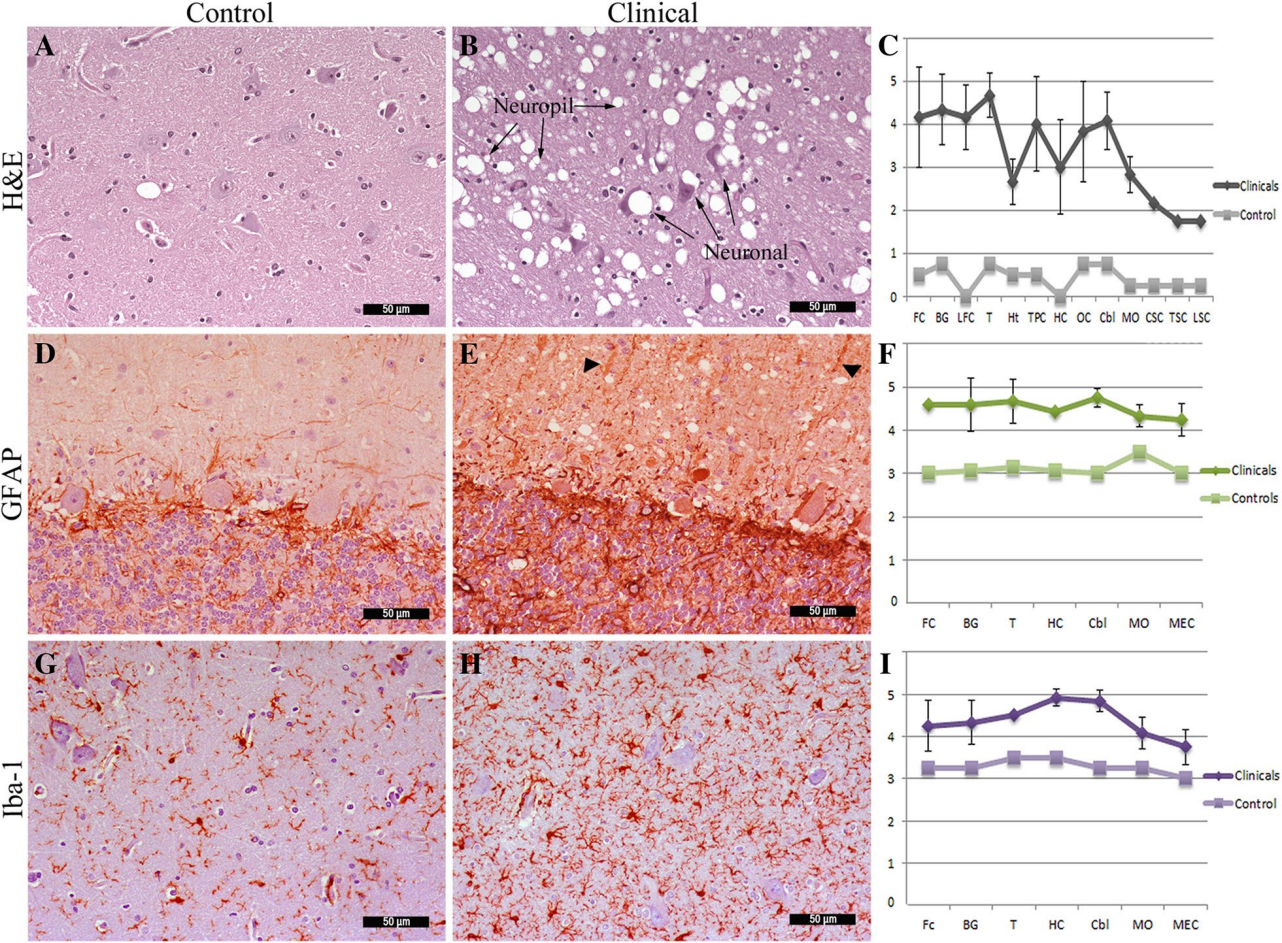
**Histopathological and immunohistochemical features (A, B, D, E, G, H) and corresponding scoring (C, F, I) in brain sections from experimental pigs. A** H&E staining, single neuropil vacuole in thalamus of control pig, **B** neuropil and intraneuronal vacuolation in Sh-BSE pig, and **C** comparative scoring. GFAP staining: **D** cerebellar cortex of control pig, **E** increased astrocytic reaction in clinically affected pig, and **F** comparative scoring. Iba-1 staining: **G** hippocampus of control pig, **H** increased number of reactive microglia in clinical pig, and **I** comparative scoring. Scores represent the mean ± standard error. Areas: FC frontal cortex, BG basal ganglia, LFC lateral frontal cortex, T thalamus, Ht hypothalamus, TPC temporal/parietal cortex, HC hippocampus, OC occipital cortex, Cbl cerebellum, MO medulla oblongata, CSC cervical, TSC thoracic, LSC lumbar spinal cord.

Microscopic evaluation revealed occasional neuropil vacuolation in the molecular layer of the cerebral and cerebellar cortex, thalamus/hypothalamus (Figure 1A) and white matter of different brain sections of the control and preclinical disease stage pigs. Unlike in other species, the incidental vacuolation in the pig cannot be unequivocally differentiated from TSE specific vacuoles, a lesion profile from the negative control is also presented for comparison. Large intraneuronal and neuropil vacuolation (spongiform changes) and increased glial cell reaction were observed only in the CNS of pigs that developed neurological signs.

Five of the 6 clinically affected pigs showed severe neuropil and neuronal vacuolation in the cerebral cortex. This effect was milder in P-2. Lesions were more severe in frontal versus occipital areas and were severe in deeper layers of the cerebral cortex. In the basal ganglia, severe neuropil vacuolation was observed in the caudate nucleus in all clinical animals. Neuronal vacuolation in the septum was moderate in 5 pigs and mild in one case (P-1). In hippocampal regions CA1 and CA3 neuronal vacuolation was severe in P-1 and P-3, and mild in the remaining 4 pigs. Occasional neuropil vacuolation of the gyrus dentatus was observed in P-1 and P-3.

Lesions decreased in intensity in a rostro-caudal direction in the brain stem. All pigs showed extreme vacuolation in the thalamus (Figure 1B) and relatively mild vacuolation in the hypothalamus. Moderate-to-severe neuropil vacuolation and a mild-to-moderate neuronal vacuolation were observed in the mesencephalon of P-2, P-4 and P-6, particularly in the red nuclei, trigeminal and facial medullary nuclei. Occasional mild spongiosis was detected in the oculomotor nuclei. In the medulla oblongata, mild-to-moderate spongiosis was observed in the HMN, RF and ON and moderate-to-severe spongiosis in the DMNV and NTN. Occasional neuronal vacuoles were observed in the HMN, NTN, ON and RF and mild vacuolation in the DMNV. In all pigs the dorsal and ventral horns of the spinal cord exhibited occasional neuronal and neuropil vacuolation. All lobes of the cerebellar cortex showed severe vacuolation, especially the molecular and granule-cell layers. Histopathological differences between control and affected animals are shown in Figure 1C.

GFAP immunostaining in the control pig (P-8) revealed non-reactive astrocytes, mainly in white matter, mild staining of fine fibers in the grey matter, and moderate ependymal and perivascular staining in all sections. In the HC, GFAP staining was observed in the molecular layer adjacent to the granular cell layer in the fascia dentata. Occasional-to-mild staining in the granular and molecular layers was also seen in the control cerebellum (Figure 1D). P-8 showed a mild increase in GFAP staining. A widespread and marked increase in GFAP expression was observed in the brains of clinically-affected pigs. This increase was due to the greater abundance of large size astrocytes in the gray and white matter, mainly in the FC, BG, T and Cbl. Immunolabelling was also observed in the prolongations of Bergmann glia of the molecular layer of the cerebellum (Figure 1E). Differences in the distribution of GFAP staining between control and Sh-BSE brain sections are shown in Figure 1F.

Microglia was analyzed by specific Iba-1 IHC, which revealed immunolabelling in the white matter in various sections of the negative control pig (Figure 1G). An abundance of microglia with much larger round or amoeboid cell bodies was observed in the brain sections of clinically affected pigs (Figure 1H). The increase in Iba-1 expression was particularly marked in the T, HC and Cbl, and less immunolabeling in the spinal cord (Figure 1I).

**Figure 2:**
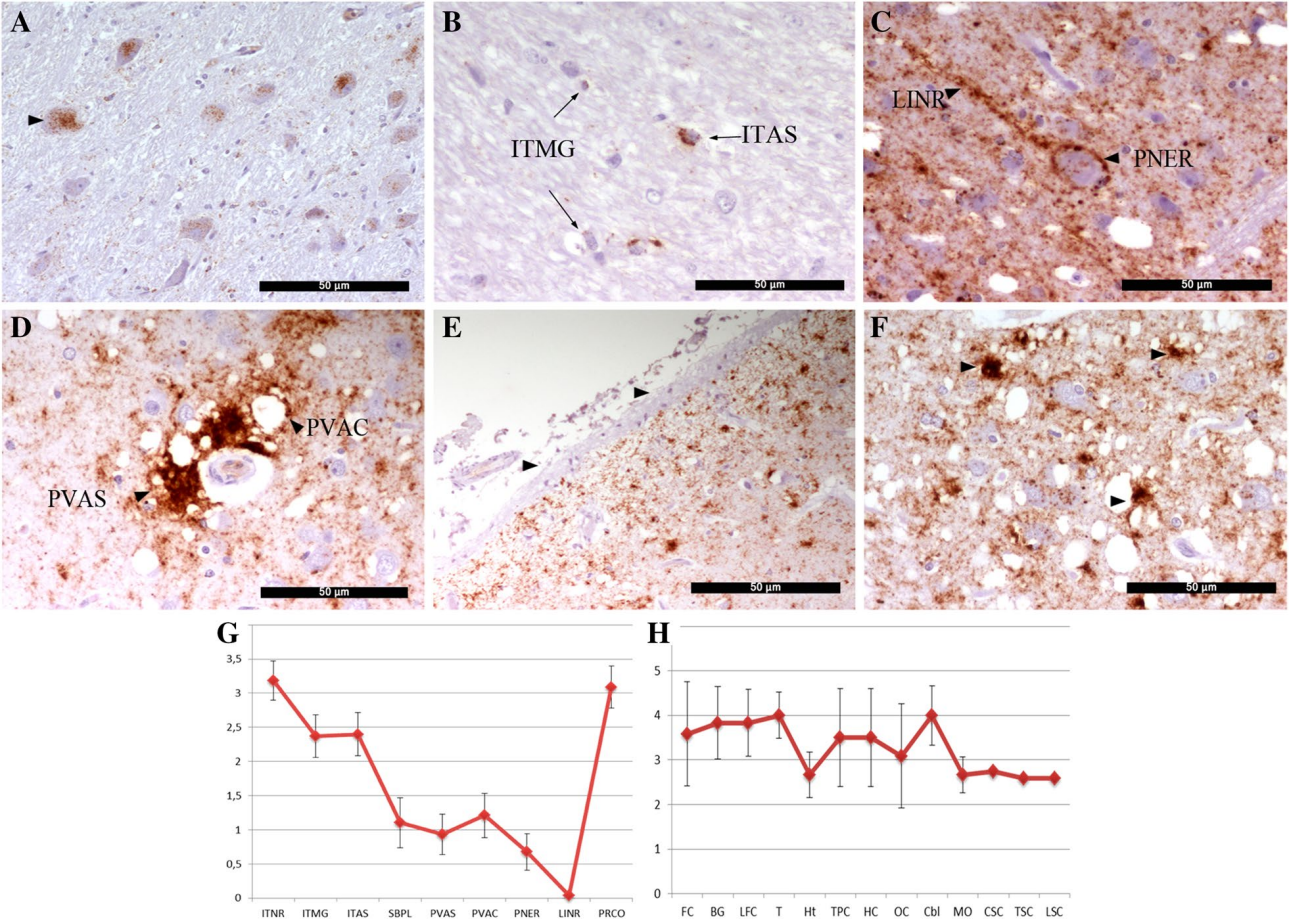
**PrP** ^**Sc**^ **types and distribution pattern in different sections of the CNS of clinically affected pigs. A** Intraneuronal (ITNR) type, **B** intra-astrocytic (ITAS) and intra-microglial (ITMG) type, **C** perineuronal (PNER) and linear (LINR) type, **D** perivascular (PVAS) and perivacuolar (PVAC) types, **E** subpial (SBPL) type, **F** particulate/coalescing (PRCO) showing amorphous aggregations (arrowheads). **G** Scoring PrP^Sc^ profile of the deposition types. **H** PrP^Sc^ distribution in CNS. Scores represent the mean ± standard error. Areas: FC frontal cortex, BG basal ganglia, LFC lateral frontal cortex, T thalamus, Ht hypothalamus, TPC temporal/parietal cortex, HC hippocampus, OC occipital cortex, Cbl cerebellum, MO medulla oblongata, CSC cervical, TSC thoracic, LSC lumbar spinal cord.

IHC revealed no PrP^Sc^ deposition in the CNS of the control and preclinical animals. Nine different deposition types were clearly identified in the CNS of the clinically affected pigs and confirmed the diagnosis of a TSE in these animals. The PrP^Sc^ types found were as follows:

*Intraneuronal type* (ITNR): accumulation of fine to coarse granular deposits of PrP^Sc^ in the neuronal perikarya surrounding the nucleus (Figure 2A). This pattern was especially observed in different sections of the spinal cord, DMNV, NTN and ON nuclei in medulla oblongata, thalamus and hippocampus.

*Intra*-*astrocytic type* (ITAS): this type was expressed by multiple small granules scattered in the cytoplasm of astrocyte-resembling cells.

*Intra*-*microglial type* (ITMG): characterized by one as single or a few large granules in close proximity to microglia-like nuclei.

Both ITAS and ITMG were found in all the sections, especially in thalamus and hippocampus, but well identified in the white matter of cerebral (Figure 2B) and cerebellar cortex, although in less amounts than in the grey matter.

*Perineuronal type* (PNER): thin deposits of PrP^Sc^ around the plasmalemma of the neuronal bodies observed in moderated levels in different basal ganglia nuclei (Figure 2C).

*Linear type* (LINR): thick thread-like deposits of PrP^Sc^ in the neuropil occasionally present in the basal ganglia (Figure 2C) and hippocampus associated to PRCO type.

*Perivascular type* (PRVS): thick, strongly labeled PrP^Sc^ accumulation around blood vessels located in the gray matter of the cerebral cortices (Figure 2D).

*Perivaculoar type* (PRVC): PrP^Sc^ deposits surrounding vacuolar lesions were detected in cerebral (Figure 2D) and cerebellar cortex, basal ganglia and thalamus in all the pigs.

*Subpial type* (SBPL): continuous loose mesh of PrP^Sc^ accumulation underneath the pia mater, especially in the cerebral (Figure 2E) and cerebellar cortex.

*Particulate to coalescing type* (PRCO): Conspicuous deposits in the neuropil associated with amorphous masses of PrP^Sc^ were observed in the gray matter of the cerebral cortex (Figure 2F), it was diffuse in 5 pigs and multifocal in P-4. This pattern was also observed in the cerebellar cortex, basal ganglia, thalamus/hypothalamus and hippocampus. Fine particles were observed in the septal and caudate nuclei in basal ganglia.

The predominant PrP^Sc^ types present in the CNS of all the pigs were the ITNR and the PRCO, while the LINR type was the less observed (Figure 2G).

The PrP^Sc^ distribution pattern along the different sections of the CNS revealed that the higher deposits were observed in the cerebral cortex; thalamus and cerebellum, whilst the lowest depositions were in the hypothalamus and spinal cord (Figure 2H).

The global Spearman correlation values for histopathology and immunohistochemistry in all samples are shown in Table 2. Spongiform changes were significantly correlated (*P* < 0.001) with PrP^Sc^ deposition. Despite no correlation between spongiform changes and glial reactivity, PrP^Sc^ deposition was significantly correlated with GFAP and Iba-1 immunostaining. Moreover, a significant correlation was observed between GFAP and Iba-1 immunostaining (*P* < 0.001).

**Table 2:** **Spearman correlation values between histological features (vacuolation and PrP** ^**Sc**^ **; GFAP and Iba-1) in all groups.**

Spearman’s rho	PrP^Sc^	GFAP	Iba-1
Vacuolation	856 (***)		
PrP^Sc^		331 (**)	316 (**)
GFAP			912 (***)

Correlations were estimated using the full set of data obtained from all CNS sections (global correlation)

*Empty cells* No correlation or duplicated results

** Correlation is very significant at the 0.01 level

*** Correlation is highly significant at the 0.001 level

### Histopathology and PrP^Sc^ detection in the retina

**Figure 3:**
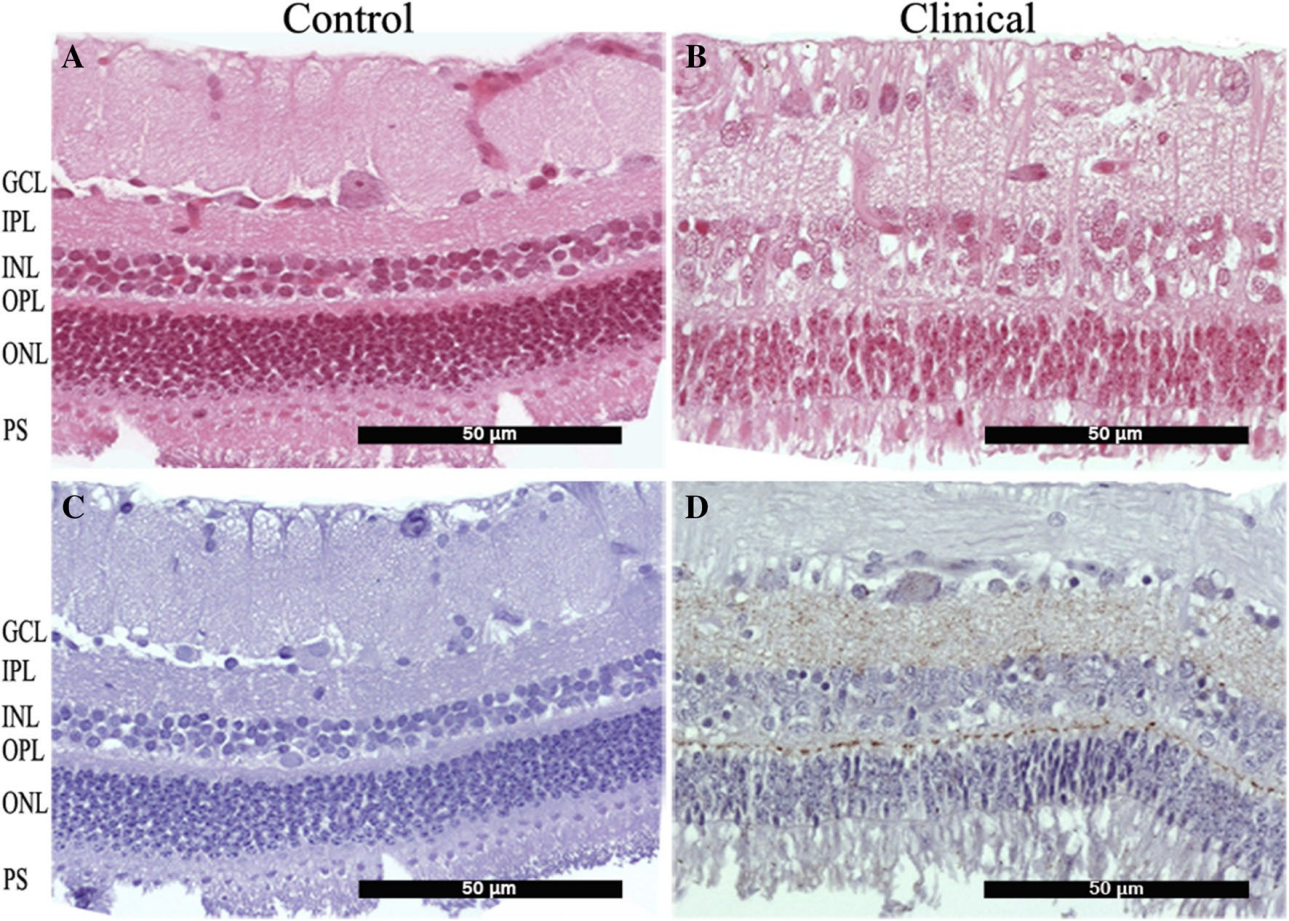
**Photomicrographs of retinal cross-sections from negative control (A, C) and clinically affected pigs (B, D).** All layers of the control retina (**A**) show normal organization and cytoarchitecture. GCL ganglion cell layer, IPL inner plexiform layer, INL inner nuclear layer, OPL outer plexiform layer, ONL outer nuclear layer, PS photoreceptor segments. **B** Moderate disorganization and decreased cellular density is observed within the INL and ONL in clinically affected pigs. **C** Absence of PrP^Sc^ staining in control retina. **D** Granular intra- and extraneuronal deposition in GCL, IPL and OPL of Sh-BSE pig.

The control pig showed no histopathological changes in the optic nerve or retina (Figure 3A). However, clinically affected pigs showed histological lesions of the retina of varying degrees of severity. Histopathological findings included loss of outer limiting layer definition, outer plexiform layer (OPL) atrophy, and disorganization and loss of nuclei of both outer (ONL) and inner nuclear layers (INL). A thickening of the photoreceptor layer (PS) was also observed, a result of the disorganization and elongation of the photoreceptor segments (Figure 3B). While no PrP^Sc^ was detected in the optic nerve or retina (Figure 3C) of the control pig, occasional granular PrP^Sc^ deposition was observed in the optic nerve of five clinically affected pigs. PrP^Sc^ was also observed in the neuroretina of all clinically affected pigs in the ganglion cell layer, OPL and IPL. The deposition pattern was granular in the plexiform layers of the retina and intraneuronal in the ganglion cell layer (Figure 3D). PrP^Sc^ was not detected in any ocular tissues other than the neuroretina.

### Western blot analysis

**Figure 4:**
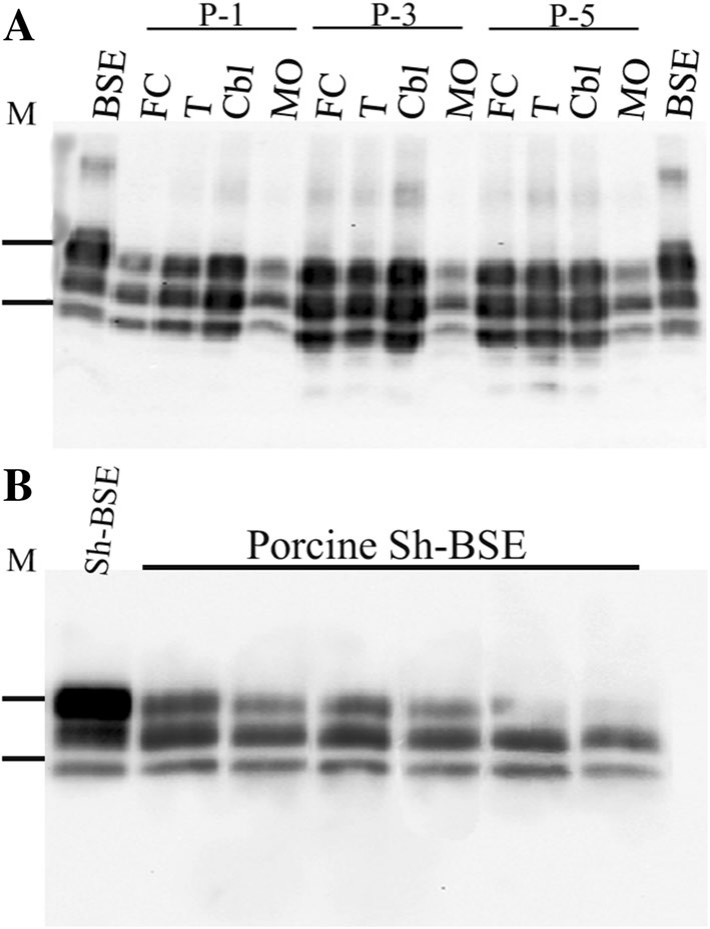
**Sh-BSE glycosylation pattern in pigs.** **A** Minigel: PrP^res^ in frontal cortex (FC) thalamus (T) cerebellum (Cbl) and medulla oblongata (MO) of 3 clinically affected pigs. **B** Bio-Rad gel: molecular signature of bovine spongiform encephalopathy after passage in sheep (Sh-BSE) and of sheep-BSE in pigs. Note the difference in PrP^res^ glycosylation in the original sheep BSE versus porcine sheep-BSE. No differences were observed in the molecular masses of the unglycosylated forms of PrP^res^. M, are 20.1 and 29.1 kD molecular mass markers.

Western blot revealed no PrP^res^ in the CNS of preclinical (P-7) and control (P-8) pigs. However, brain sections from clinically affected pigs showed a characteristic 3-band pattern. The medulla oblongata band signal appeared to be less intense than the frontal cortex, thalamus and cerebellum samples (Figure 4A). Differences in the molecular signature between the original inoculum (Sh-BSE) and sheep-BSE in pigs were observed. The molecular mass of the diglycosylated band of Sh-BSE was higher than that of porcine Sh-BSE, which produced a predominant monoglycosylated band. By contrast, the molecular masses of the unglycosylated PrP^res^ were similar (Figure 4B).

### Immunoassay analysis

The results of IDEXX revealed optical density (OD) values of 0.03 in the negative control and 3.695–4.349 in the CNS of clinically affected pigs (Table 3).

**Table 3:** **PrP** ^**Sc**^ **detection by immunohistochemistry (IHC) and IDEXX in the central nervous system (CNS), peripheral nervous system (PNS), lympho-reticular (LRS), gastrointestinal tract (GIT), skeletal muscles (SM) and other organs of individual pigs.**

SITE	Tissue	P1		P2		P3		P4		P5		P6		P8	
		IHC	EIA	IHC	EIA	IHC	EIA	IHC	EIA	IHC	EIA	IHC	EIA	IHC	EIA
CNS	Brain	+	4.22	+	3.93	+	3.69	+	3.75	+	3.90	+	4.34	–	0.03
	Retina	+	ND	+	ND	+	ND	+	ND	+	ND	+	ND	–	ND
	Optic nerve	+	ND	+	ND	+	ND	+	ND	+	ND	+	ND	–	ND
PNS	Vagus nerve	+	ND	+	ND	+	ND	+	ND	+	ND	+	ND	–	–
	Brachial nerve	–	–	+	0.20	+	0.17	–	–	–	–	–	–	–	–
	Sciatic nerve	+	0.43	+	0.30	+	0.28	+	0.29	+	0.34	+	0.16	–	–
LRS	Tonsil	+	0.17	+	0.08	–	–	+	0.15	–	–	–	–	–	–
	Retropharyngeal ln.	–	–	–	–	–	–	–	–	–	–	–	–	–	–
	Submandibular ln.	–	–	–	0.35	–	–	–	–	–	–	–	–	–	–
	Prescapular ln.	–	–	–	–	–	–	–	–	–	–	–	–	–	–
	Mediastinal ln.	+	0.16	–	–	–	–	–	–	–	–	–	–	–	–
	Spleen	–	–	–	–	–	–	–	–	–	–	–	–	–	–
	Mesenterial ln.	–	–	+	0.23	–	–	+	0.19	+	0.54	–	–	–	–
	Popliteal ln.	–	–	–	–	–	–	–	–	–	–	–	–	–	–
GIT	Oesophagus	–	–	–	–	–	–	–	–	–	–	–	–	–	–
	Stomach	+	–	–	–	–	–	–	–	–	–	+	–	–	–
	Duodenum	++	–	–	–	–	–	++	–	–	–	+	–	–	–
	Jejunum	++	0.24	–	–	+	–	++	–	–	–	+	–	–	–
	Ileum	+++	0.40	+++	0.72	++	0.22	+++	1.48	+	0.23	++	0.31	–	–
	Caecum	+	–	–	–	–	–	–	–	–	–	–	–	–	–
	Colon	+	–	–	–	–	–	–	–	–	–	–	–	–	–
	Rectum	+	–	–	–	–	–	–	–	–	–	–	–	–	–
SM	Oculomotor	+	1.00	–	–	–	–	+	2.83	–	0.67	–	–	–	–
	Brachial biceps	–	–	–	–	–	–	–	–	–	–	–	–	–	–
	Semitendinosus	–	–	–	–	–	–	–	–	–	0.20	–	–	–	–
Other	Tongue	–	–	–	–	–	–	–	–	–	–	–	–	–	–
	Olfactory mucosa	–	–	–	–	–	–	–	–	–	–	–	–	–	–
	Heart	–	–	–	–	–	–	–	–	–	–	–	–	–	–
	Lung	–	–	–	–	–	–	–	–	–	–	–	–	–	–
	Liver	–	–	–	–	–	–	–	–	–	–	–	–	–	–
	Adrenal gland	–	–	–	–	+	0.15	–	–	+	0.19	–	–	–	–
	Urinary bladder	–	–	–	–	–	–	–	–	–	–	–	–	–	–
	Kidney	+	–	–	–	–	–	–	–	–	–	–	–	–	–
	Pancreas	+	–	–	–	+	–	+	0.21	+	–	+	–	–	–
	Uterus	–	–	–	–	–	–	–	–	–	–	–	–	–	–

### Histopathology, immunohistochemistry and immunoassay of the peripheral tissues

No histopathological changes were observed outside the CNS of clinically affected pigs. However, IHC and/or IDEXX revealed widespread PrP^Sc^ distribution in organs outside the CNS of these animals. Individual results of the PrP^Sc^ detection assay are provided in Table 3.

### PrP^Sc^ detection in the peripheral nervous system (PNS)

**Figure 5:**
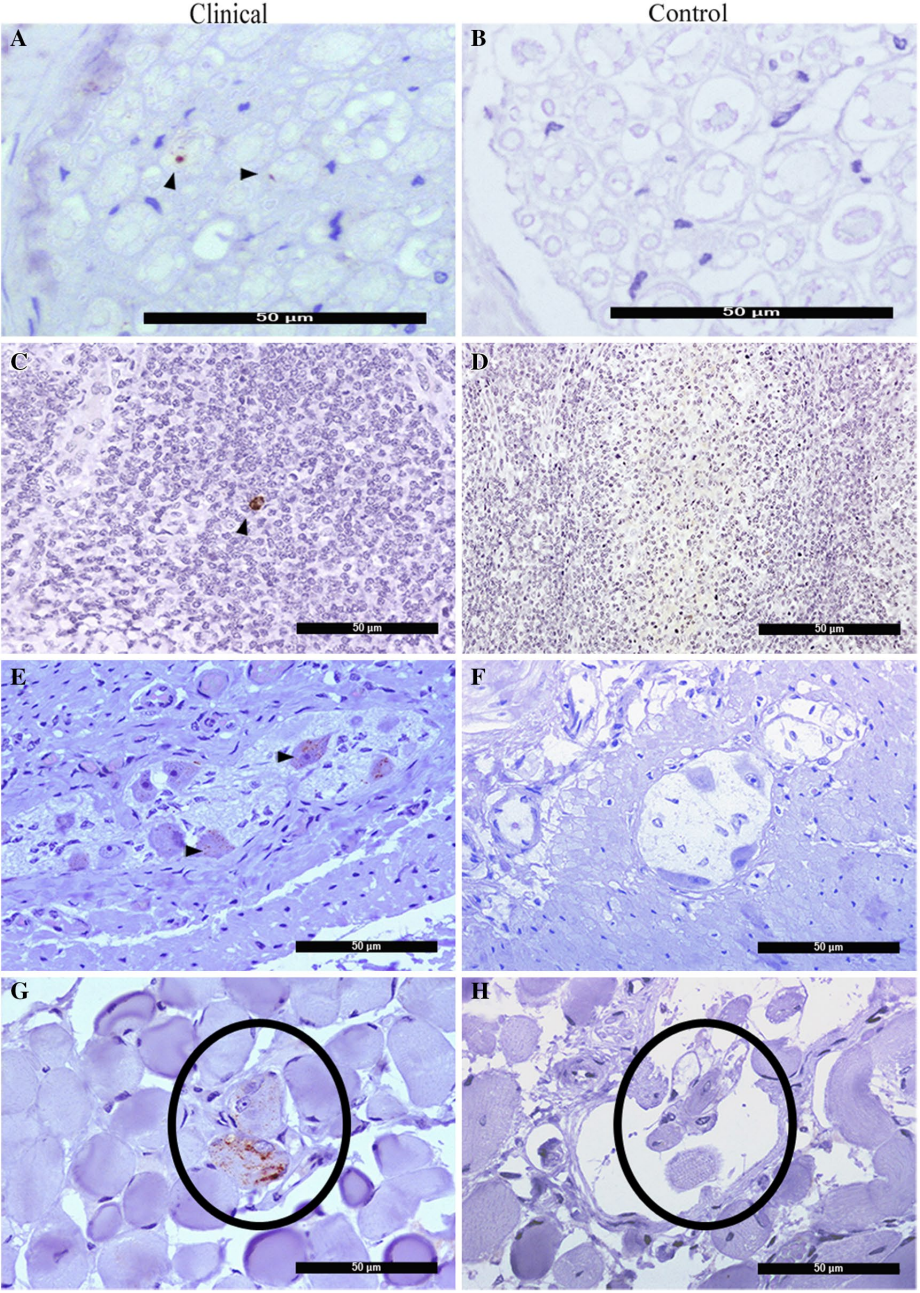
**Immunohistochemical detection of PrP** ^**Sc**^ **in peripheral tissues. A** Brachial nerve fiber bundle showing periaxonal PrP^Sc^ immunolabeling (arrowheads). **C** Tonsil showing PrP^Sc^ immunolabeling within tingible body macrophages (TBMs). **E** Intraneuronal PrP^Sc^ deposition in ileal myenteric plexus (arrow heads) in pig P-2. **G** Granular deposition in neuromuscular spindle (circle) of the oculomotor muscle of pig P-4. **B**, **D**, **F**, **H** Corresponding negative control tissues.

The vagus nerve resulted positive by the PrP^Sc^ detected in the DMNV in the MO by IHC. Both IHC and IDEXX revealed PrP^Sc^ in the brachial nerve of P-2 and P-3 with OD values ranging from 0.173 to 0.208 and in the sciatic nerve in all pigs, with OD values ranging from 0.160 to 0.430. Transversal sections of peripheral nerves showed small amounts of periaxonal labeling in some nerve fibers (Figure 5A). No neural PrP^Sc^ was detected in the control pig (Figure 5B).

### PrP^Sc^ detection in the lymphoreticular system (LRS)

In P1 and P4, PrP^Sc^ was detected in the palatine tonsil in the form of granules in tingible body macrophages (TBM) present in the lymphoid follicles (Figure 5C). This pattern was also observed in the submandibular lymph node in P-2, the mediastinal lymph node in P-1, and the mesenteric lymph node in P-2, P-4 and P-5, with OD values ranging from 0.152 to 0.545 (IDEXX). Neither IHC nor IDEXX detected PrP^Sc^ in the spleen of clinically affected or control pigs. Gut associated lymphoid tissues (GALT) were also negative in all cases. No PrP^Sc^ was observed in the lymphoid tissues of the control pig (Figure 5D).

### PrP^Sc^ detection in the gastrointestinal tract (GIT)

PrP^Sc^ deposits were consistently detected in the enteric nervous system, with granular intracytoplasmic accumulation observed in neurons of the myenteric (Auerbach’s) plexus (Figure 5E). Positivity was detected in the stomach, duodenum, jejunum, ileum, caecum, colon and rectum, with levels varying between animals. P-1, which was euthanized at 116 wpi, showed more positive sections than P-2, P-4 or P-6, which had longer incubation periods. Positive staining was also detected in the duodenum and jejunum in three and four pigs, respectively, and in the caecum, colon and rectum in one of the six pigs. Both methods revealed positive staining in the distal ileum in all pigs. This finding suggests that the ileum is the first part of the GIT to be reached by prions. No PrP^Sc^ accumulation was observed in the negative control or preclinical pigs (Figure 5F).

### PrP^Sc^ detection in skeletal muscle

Both assay methods detected PrP^Sc^ in skeletal muscle samples from oculomotor muscle in P-1 and P-4. IHC revealed granular PrP^Sc^ deposit restricted to the muscle spindles (Figure 5G); PrP^Sc^ immunolabeling was not detected in myofibrils, intramuscular nerve fascicles or in most ganglia in the muscle samples analyzed. PrP^Sc^ was detected in oculomotor muscle and semitendinosus muscle in P-5 by IDEXX only. No PrP^Sc^ accumulation was detected by either method in muscle samples from control and preclinical pigs (Figure 5H).

### PrP^Sc^ detection in the pancreas

**Figure 6:**
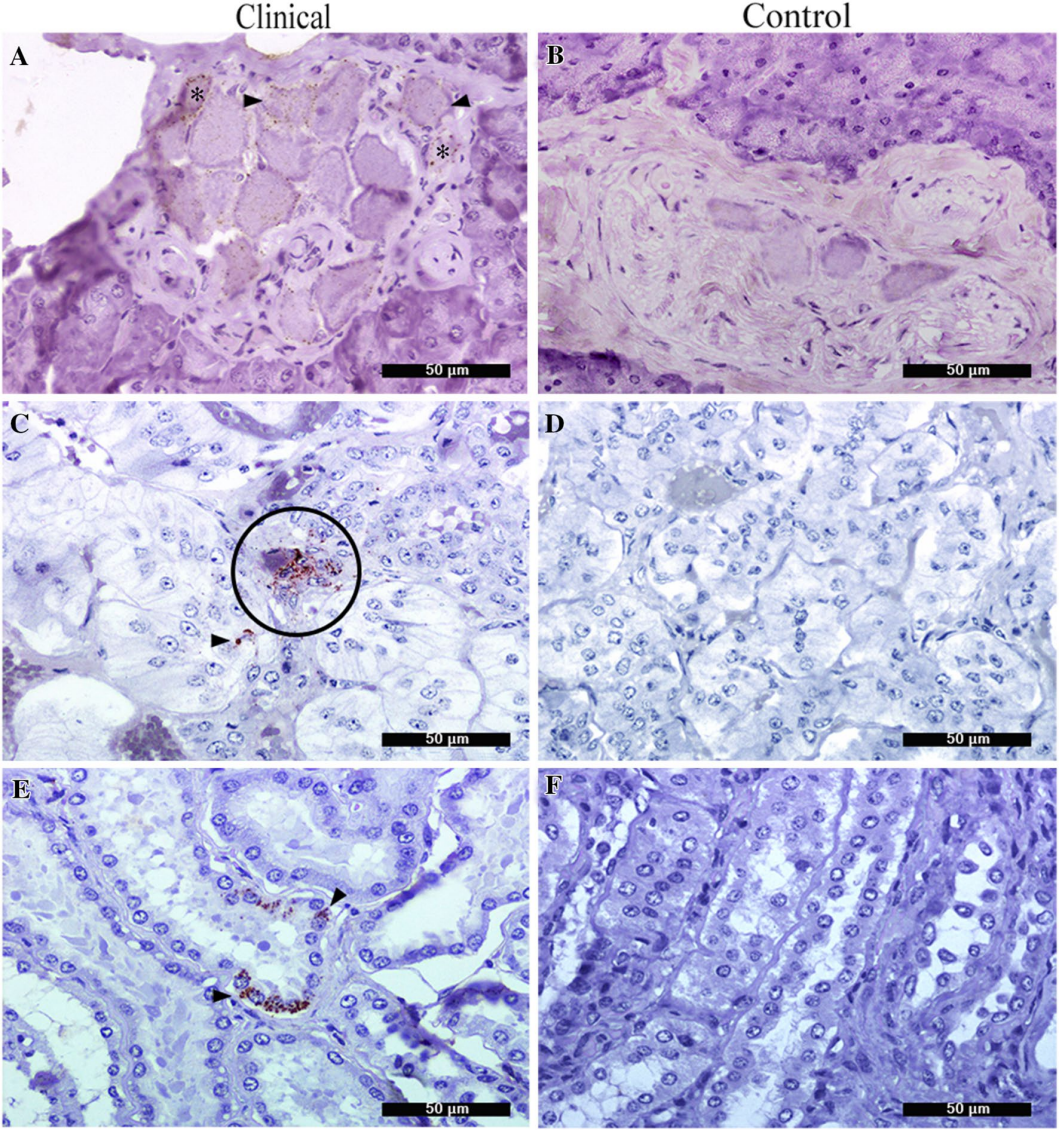
**Immunohistochemical detection of PrP** ^**Sc**^ **in peripheral tissues.** **A** Intrapancreatic ganglia neurons showing perineuronal (*) and granular intracytoplasmic labeling (arrow). **C** Adrenal gland showing granular deposits in ganglionic neurons (red circle) and chromaffin cells (arrow). **E** Sh-BSE kidney showing PrP^Sc^ deposits in tubular structure (arrow head). **B**, **D**, **E** Absence of a detectable positive signal in corresponding tissues from the control pig.

PrP^Sc^ was detected in the pancreas of five clinically affected pigs by IHC only. PrP^Sc^ deposition was observed in structures of the PNS. In these samples, intracytoplasmic and perineuronal immunolabeling was observed in the parasympathetic postganglionic neurons in pancreatic tissue (Figure 6A). No PrP^Sc^ was detected in P-2 or in the control pig (Figure 6B). No pancreatic PrP^Sc^ was detected in any of the pigs analyzed using the IDEXX technique.

### PrP^Sc^ detection in the adrenal gland

PrP^Sc^ was detected in the medullar region of the adrenal gland of P-2 in the form of granular intracytoplasmic deposits in ganglionic neurons and chromaffin cells (Figure 6C). IDEXX revealed an OD value of 0.150. However, IHC revealed no clear positivity for PrP^Sc^ in P-5, for which IDEXX determined an OD of 0.191. No PrP^Sc^ was detected in the adrenal gland of any other clinically affected or control pigs (Figure 6D).

### PrP^Sc^ detection in the kidney

Granular PrP^Sc^ deposition was observed exclusively within the epithelial tubular cells of the convoluted tubes and collecting ducts in P-1 (Figure 6E). This result was not consistent with the IDEXX result, which was negative. The kidneys of the remaining clinically affected and control pigs were negative in both assays (Figure 6F).

## Discussion

This study was aimed at investigating the susceptibility and neuropathological features of pigs intracerebrally inoculated with the BSE agent after passage in sheep, as well as describing the PrP^Sc^ distribution in peripheral tissues in this species.

In the present study, seven pigs were intracerebrally inoculated with 0.5 mL of 10% Sh-BSE homogenate. Except in one animal (P-7), which was euthanized for preclinical analysis, the transmission rate was 100%, with an incubation period range of 77–109 wpi. Two previous studies in which bovine BSE has been transmitted to pigs, reported 87.5% and 20% of rate attacks, with incubation period ranges of 74–163 and 148–175 wpi, respectively [19, 20]. Sh-BSE infected pigs show slightly shorter incubation periods. However, it is not possible to compare the incubation period of our inoculated pigs with respect to the incubation period found in the studies mentioned above, due to the lack of titration of the original inoculum. Moreover, the incubation period could also be modified in TSE due to the species barrier, which is modulated by specific polymorphisms of the PRNP gene and plays a key role in susceptibility to prion disease in other species such as sheep [35, 36], and goats [37]. Although some studies show that there are no differences in the sequence of the porcine PRNP gene [38–40], the possibility of changes in other regions of the gene or the involvement of other genes in the incubation periods of BSE in pigs should not be excluded. In addition, the restricted number of animals used does not allow comparing difference on rate attacks in previous studies with the present report. However, transmissible studies in porcine PRNP transgenic mice (Tgpo) has demonstrated that the Sh-BSE agent reached rate attack of 100% and lower survival time when compared to the original bovine BSE (19%) and other BSE isolates at first passage [15]. At two subsequent passages, the transmission rate of both Sh-BSE and bovine BSE was reported to be similar (100%) but always with a lower survival time of the Sh-BSE infected mice [15]. Recent studies have demonstrated an increase in the PrP-converting potency of Sh-BSE caused by decreases in polymorphism barriers [24] and other specific cellular factors [25], allowing Sh-BSE to be transmitted more efficiently than cattle BSE to other species [16, 17] including supposedly less susceptible hosts such as pigs [15]. The current study was in agreement with previous reports [20–22] involving intracerebral inoculation of BSE prions to pigs demonstrating that this species is susceptible to BSE. However, it is still unknown if pigs can succumb to BSE after oral exposure which is the most likely route of inoculation under natural conditions.

The clinical signs observed in the present study were similar to those described in BSE-infected pigs [20]. Animals initially showed progressive confusion, followed by motor deficits [19]. The behavioral and sensory changes were also consistent with those observed in cattle infected naturally [41] and experimentally with BSE [12]. The minimal neuropil vacuoles found in the control pig are in total agreement with previous studies [21] and apparently does not represent a clinical significant change [20]. The main pathological changes observed were neuropil spongiosis, intraneuronal vacuolation and PrP^Sc^ deposition, all of which are characteristic of TSE [41]. The lesion distribution pattern resembled that described previously in experimentally BSE- infected pigs [21] and cattle [12]; the thalamus was the most affected area, followed by the cerebellar and cerebral cortices, with the mildest effect observed in the spinal cord. PrP^Sc^ deposits were identified in the CNS of all clinically affected pigs. PrP^Sc^ deposits were typically associated with lesions in the fourth and fifth layers of the cerebral cortex. Intracellular (ITNR, ITAS and ITMG) and particulate/coalescing type PrP^Sc^ deposition were the most commonly observed patterns in the different CNS samples, in line with previous findings in sheep [29, 30] and pigs [21] experimentally infected with BSE. Similarities in the PrP^Sc^ deposition types and distribution pattern could be explained by the high stability of the BSE agent reported for different breeds and different genotypes of the prion protein gene (PRNP) in sheep [30]. In addition, the porcine PRNP gene has been described to be very homogenous [38–40].

The glial reaction in all affected pigs was characterized by marked astrocytosis and microgliosis. Astrocytosis was diffusely distributed throughout the brain of affected pigs, perhaps caused by the accumulation of PrP^Sc^ or by cytokines secreted from astroglial or microglial cells [42]. Microgliosis was present in the deeper layers of the gray matter in the cerebral cortex, which also showed vacuolation and PrP^Sc^ deposition, in accordance with previous findings in mice [43]. The most extreme microglial activation was observed in the hippocampus of all affected pigs, as previously described for CJD [44]. Numerous astrocytic processes and reactive microglia have been described in pigs experimentally infected with BSE [45]. Our results suggest that astrogliosis and microgliosis are common neuropathological features of Sh-BSE infection in pigs, as described for TSE in other species [28, 43, 45–47].

Histopathological changes indicative of retinal degeneration were observed in all clinically affected pigs. This has not been previously described in pigs experimentally infected with BSE. Neuronal vacuolation in the GCL and disorganization in the plexiform and nuclear layers have been reported in both experimental [48] and natural scrapie infections in sheep [49] and goats [50], chronic wasting disease (CWD) in mule deer [51] and in CJD-infected mice [52]. IHC revealed higher levels of PrP^Sc^ in the retina than in the optic nerve, where staining was less intense and more irregularly distributed, as described in both sCJD and nvCJD [53]. The presence of PrP^Sc^ in the optic nerve and retina is consistent with the centrifugal spread of the agent from the brain, presumably via the optic nerve [52]. This may indicate the existence of other routes of PrP^Sc^ migration to the retina (e.g., via the extracellular space [54], the ad-axonal route along the optic nerve, or both [55]). Other authors have suggested that the spread occurs from the subarachnoid space into the perineural space of the optic nerve, and from there to the epichoroidal and episcleral tissues of the eyeball [56]. Alternatively, the increased presence of PrP^Sc^ in the retina more than in the optic nerve could be attributed to the higher presence of PrP^c^ in the membranes of retinal neurons. Our detection of PrP^Sc^ in different retinal layers is in accordance with previous observations in TSE in mice [52], feline spongiform encephalopathy (FSE) [57], scrapie [49], CWD [51], BSE [56] and in patients with sporadic and nvCJD [53].

Western blot revealed a characteristic 3-band pattern that clearly differed from the original inoculum, with a predominant monoclycosylated band. This finding is consistent with previous Western blot findings in BSE-infected pigs [58]. Our results reinforce the hypothesis that this particular signature is associated with the porcine PrP^c^ properties described in Tgpo mice [15].

The IDEXX enzyme immunoassay, which is not validated for PrP^sc^ in pigs, detected PrP^Sc^ in samples that tested positive in other postmortem assays, but detected no PrP^sc^ in negative control tissues. Analysis of peripheral tissues revealed widespread dissemination of PrP^Sc^ in many organs other than the CNS. This finding suggests that unlike in cattle where BSE is confined mainly in the nervous system, in the pig, BSE prions can propagate in peripheral tissues as reported in sheep [59–61]. However, it is not possible to ascertain that the peripheral distribution of the agent is due to centrifugal dissemination from the brain through the nerves as it is also probable that during an ic challenge part of the inoculum enters into the blood circulation and can be disseminated to the periphery where it can propagate in target tissues [62].

PrP^Sc^ deposition in brachial and sciatic nerves has also been described in cattle experimentally infected with L-type BSE [63] and in BSE-infected sheep [64].

Immunohistochemistry demonstrated the presence of PrP^Sc^ in the lymphoreticular system of our Sh-BSE infected pigs. The assay revealed sporadic intracytoplasmic accumulation within the tingible body macrophages in some lymph nodes, findings that were subsequently corroborated by IDEXX, in good agreement with previous findings in sheep experimentally infected with BSE [64]. In contrast to our findings, previous studies reported no infectivity of lymphoid tissues in BSE-infected pigs [19]. No PrP^Sc^ was detected in the spleen or GALT of our pigs, in line with previous studies of BSE-infected cattle [65] and FSE [57].

PrP^Sc^ accumulation in the gastrointestinal tract of Sh-BSE infected pigs has not been described in similar experiments using this species. We observed PrP^Sc^ deposition in the myenteric plexi without apparent morphological alterations of the enteric neurons, as seen in cattle experimentally infected with BSE [65]. This finding is indicative of a potential centrifugal spread of the Sh-BSE agent from the CNS via the vagus nerve to the peripheral nervous system, and may account for the large deposits of PrP^Sc^ observed in the dorsal motor nucleus of the vagus nerve in the medulla oblongata.

We observed PrP^Sc^ deposition in nerve fibers of the oculomotor muscle in two pigs. In cattle naturally infected with BSE [66], PrP^Sc^ has been detected in intramuscular nerve fibers and muscle spindles. Although we found no PrP^Sc^ in the oculomotor muscle of any other clinically affected pigs, positive labeling was observed in the oculomotor nuclei in the mesencephalon of all clinically affected pigs.

Pancreatic PrP^Sc^ staining was observed in 5 pigs. Analysis of pancreatic nervous tissue has revealed PrP^Sc^ deposition in the islets of Langerhans in natural scrapie [34]. In natural BSE [56], PrP^Sc^ deposition has been documented in the nerve fibers of the adrenal gland. In agreement with previous findings in natural scrapie [34], one pig showed PrP^Sc^ immunolabeling in the medullary region of the adrenal gland, associated with chromaffin cells, which are considered modified sympathetic postganglionic neurons. Similarly, the presence of PrP^Sc^ within the epithelial tubular cells of the convoluted tubules and the collecting ducts in the kidney in one pig has been described in FSE [67], suggesting possible prionuria.

In addition to the large amount of PrP^Sc^ observed in the CNS of Sh-BSE-infected pigs, PrP^Sc^ was widely distributed in the peripheral tissues, although the extent of this distribution varied between animals. This variation may be related to the distribution of PrP^Sc^ within individual organs, the exact anatomical location points at which samples were collected, and the detection limits of the techniques used. More sensitive studies, such as in vitro protein misfolding cyclic amplification (PMCA) and mouse bioassays will be needed to clarify the distribution and infectivity of PrP^Sc^ in peripheral tissues of Sh-BSE infected pigs. These assays will most likely indicate a higher number of PrP^Sc^-positive peripheral organs.

Comparison with previous studies of cattle-BSE in pigs revealed that the incubation period of Sh-BSE in our pigs was generally shorter [20, 21] and that PrP^Sc^ was present in more peripheral tissue types [19]. We believe that these differences may be due to a modification in the pathogenicity of the cattle-BSE agent caused by its prior passage in sheep, as previously described in TgPo mice [15]. However, studies of natural routes of transmission (e.g., oral) will be required to determine the real susceptibility of pigs to the Sh-BSE agent.

## Abbreviations

BG: basal ganglia
BSE: bovine spongiform encephalopathy
PrP^C^: cellular prion protein
CNS: central nervous system
Cbl: cerebellum
CSC: cervical spinal cord
CJD: Creutzfeldt-Jakob Disease
DMNV: dorsal motor nucleus of the vagus nerve,
FSE: feline spongiform encephalopathy
FC: frontal cortex
GIT: gastrointestinal tract
GFAP: glial fibrillary acidic protein
GALT: gut associated lymphoid tissues
H&E: hematoxylin and eosin
HC: hippocampus
HMN: hypoglossal motor nucleus
Ht: hypothalamus
IHC: Immunohistochemistry
INL: inner nuclear layers
ITAS: intra-astrocytic
ITNR: intraneuronal
ITMG: intra-microglial
LFC: lateral frontal cortex
LINR: linear,
LSC: lumbar spinal cord
LRS: lympho-reticular system
MO: medulla oblongata
Ms: mesencephalon
NFP: neuropilo fine punctate
nvCJD: new variant of Creutzfeldt-Jakob disease
NTN: nucleus of the trigeminal nerve spinal tract
OC: occipital cortex
ON: olivary nucleus
OD: optical density
ONL: outer nuclear layers
OPL: outer plexiform layer
PRCO: particulate/coalescing
PrP^Sc^: pathological Prion Protein
PNER: perineuronal
PNS: peripheral nervous system
PVAC: perivacuolar
PVAS: perivascular
PS: photoreceptor layer
PRNP: prion protein gene coding
PrP: prion protein
PrP^res^: resistant prion protein
RF: reticular formation
RT: room temperature
Sh-BSE: sheep-bovine spongiform encephalopathy
sCJD: sporadic Creutzfeldt-Jakob disease
SBPL: subpial
TPC: temporal/parietal cortex
T: thalamus
TSC: thoracic spinal cord
TBM: tingible body macrophages
Tgpo: transgenic mice expressing porcine prion protein
TSE: transmissible spongiform encephalopathies
